# Multiparametric ultrasound in the detection of prostate cancer: a systematic review

**DOI:** 10.1007/s00345-015-1523-6

**Published:** 2015-03-12

**Authors:** Arnoud Postema, Massimo Mischi, Jean de la Rosette, Hessel Wijkstra

**Affiliations:** 1 Department of Urology, Academic Medical Center, Meibergdreef 9, 1105 AZ Amsterdam, The Netherlands; 2 Department of Electrical Engineering, Eindhoven University of Technology, Eindhoven, The Netherlands

**Keywords:** Prostate cancer, Multiparametric UltraSound, Doppler, Contrast-enhanced ultrasound, Elastography, Shear wave elastography, ANNA/C-TRUS, Multiparametric MRI

## Abstract

**Purpose:**

To investigate the advances and clinical results of the different ultrasound modalities and the progress in combining them into multiparametric UltraSound (mpUS).

**Methods:**

A systematic literature search on mpUS and the different ultrasound modalities included: greyscale ultrasound, computerized transrectal ultrasound, Doppler and power Doppler techniques, dynamic contrast-enhanced ultrasound and (shear wave) elastography.

**Results:**

Limited research available on combining ultrasound modalities has presented improvement in diagnostic performance. The data of two studies suggest that even adding a lower performing ultrasound modality to a better performing modality using crude methods can already improve the sensitivity by 13–51 %. The different modalities detect different tumours. No study has tried to combine ultrasound modalities employing a system similar to the PIRADS system used for mpMRI or more advanced classifying algorithms.

**Conclusion:**

Available evidence confirms that combining different ultrasound modalities significantly improves diagnostic performance.

## Introduction

The tools for the detection of prostate cancer (PCa) usually consist of digital rectal examination (DRE), serum prostate-specific antigen (PSA) measurement and greyscale TransRectal UltraSonography (TRUS). Since these investigations are limited in terms of sensitivity and positive predictive value (PPV), the diagnosis needs to be confirmed using TRUS-guided systematic random biopsies [1]. The resulting sample error from these untargeted biopsies is cause of many negative biopsies, while significant tumours are missed or under-graded [2].

Clearly, diagnosis through systematic untargeted biopsies is far from ideal, and the lack of an adequate imaging tool is a major deficit of the diagnostic pathway. Consequently, research is prioritized towards finding imaging techniques that enable replacing systematic untargeted biopsies by a few targeted biopsies. When a sufficiently high negative predictive value (NPV) is attained, it should be possible to exclude PCa based on imaging alone, eliminating the necessity of taking prostate biopsies in every patient with a suspicion based on DRE or PSA.

Research on PCa imaging has focussed on two platforms: magnetic resonance imaging (MRI) and ultrasound. Different MRI modalities exist. Since one modality alone does not seem to have sufficient diagnostic accuracy, the current literature recommends combining them into multiparamteric MRI (mpMRI) [3].

For the ultrasound platform too, various modalities were developed. These include dynamic contrast-enhanced UltraSound (DCE-US), colour Doppler ultrasound (CDU), power Doppler ultrasound (PDU), computerized transrectal ultrasound (C-TRUS) and elastography. Similar to the development of mpMRI, the usage of a combination of these ultrasound-based modalities, “multiparametric UltraSound (mpUS)”, could potentially improve the diagnostic performance. This paper presents the basic principles and performance of different ultrasound-based modalities and investigates the clinical results of combining them into mpUS.

## Methods

**Fig. 1:**
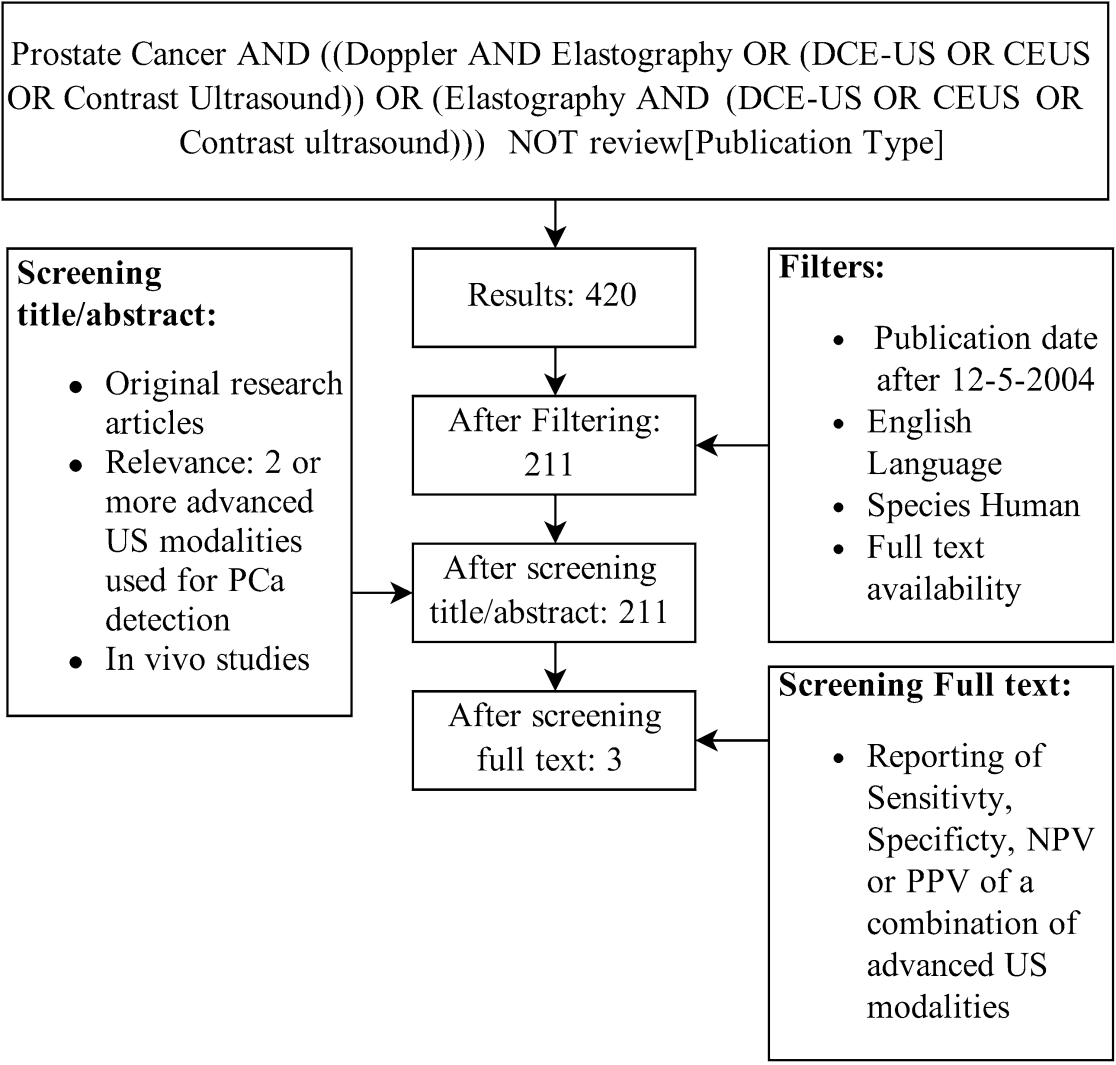
Flow chart. *PCa* prostate cancer, *US* ultrasound, *NPV* negative predictive value, *PPV* positive predictive value

A systematic literature search on mpUS was performed using the Medline database. The aim was finding original articles concerning the detection of PCa with at least two advanced ultrasound modalities (Fig. 1). The exact search term was: “Prostate Cancer AND ((Doppler AND Elastography OR (DCE-US OR CEUS OR Contrast ultrasound)) OR (Elastography AND (DCE-US OR CEUS OR Contrast ultrasound))) NOT review [Publication Type]”. Additional filters were applied to exclude articles published over 10 years ago, animal studies, non-English texts and articles with unavailable full texts. The titles and abstracts of the remaining results were screened for relevance. After full-text evaluation, studies were selected that reported the in vivo diagnostic performance of a combination of ultrasound techniques.

## Results

### Greyscale TRUS

Conventional transrectal greyscale ultrasound (GSU) is currently the standard imaging tool for the prostate [1]. GSU is used for volumetry, needle guidance for systematic biopsies and guiding seed placement in brachytherapy [4]. The sensitivity of GSU for prospective tumour detection—varying by experience—has been reported to be up to 60 %. This reflects known sonographic properties of PCa: approximately 60 % of tumours appear hypoechogenic [5]. Around 35–39 % of tumours are isoechogenic, limiting the detection potential of GSU [6, 7]. The performance reported in the literature varies widely with sensitivities ranging between 8 and 88 % and specificities ranging from 42.5 to 99 % [8–13]. The three studies with the highest specificities report the lowest sensitivities, possibly reflecting conservative interpretation [11–13]. The PPV and specificity suffer from the high number of false positives caused by processes such as prostatitis, benign prostate hyperplasia, atrophy and infarction that mimic the typical appearance of PCa [5].

### Computer-analysed TRUS

Several systems for computerized analysis of GSU images have been developed that use various algorithms to predict whether tissue is malignant [14]. The first and so far best results in clinical testing have come from the artificial neural network/C-TRUS (ANNA/C-TRUS) system. In the current “network version” of the C-TRUS system, the static TRUS images are transmitted to the C-TRUS server by a secured web-based system respecting the protection of patient data. The C-TRUS system uses an ANNA algorithm to analyse the ultrasound signals and highlight suspicious areas on the images that are transmitted back to the user [15]. An external validation study comparing results from the current version of the C-TRUS system with radical prostatectomy (RP) specimens in 28 patients showed a sensitivity, specificity, PPV and NPV of 83, 64, 80 and 63 %, respectively [16]. In the primary study that evaluated patients with up to 70 prior negative systematic biopsies, C-TRUS detected 66 cancers in 132 men in which the cancer was missed by 12 (median) systematic biopsies [17]. In a prospective study in 164 patients, C-TRUS/ANNA-guided biopsies were compared to the final pathology of the RP specimen. ANNA/C-TRUS could preoperatively predict the RP Gleason grade of the index lesion in 85 % [18]. Larger, multicentre trials are underway to support the evidence published so far [19].

The initial results for a different quantification technique, histoscanning, were favourable. Two small studies comparing histoscanning with RP specimens comprising of nine and 27 patients showed sensitivities of 90–100 % and specificities of 72–82 % [19]. However, histoscanning could not accurately predict biopsy results in a cohort of 198 men according to a sextant analysis [20]. Finally, a paper evaluated the ability of histoscanning to detect, characterize and locally stage PCa by comparing it with TRUS-guided prostate biopsies, transperineal template prostate biopsies (TTBs) and RP specimens in three separate studies [21]. In the comparison between histoscanning-targeted biopsies and standard TRUS-guided biopsies, the former had an overall cancer detection rate of 38.1 % and the latter of 62.5 %. In the comparison between the histoscanning-targeted biopsies and standard TTB, the targeted biopsies had an overall cancer detection rate of 13.4 % compared to 54.4 % for standard TTB. No correlation between total tumour volume estimates from histoscanning and RP specimens was found, and the sensitivity and specificity to detect tumour volumes over 0.5 mL were 37 and 71 %, respectively. Histoscanning is, therefore, currently not recommended to reliably identify and characterize PCa.

### Doppler/power Doppler

PCa requires angiogenesis to develop into clinically significant disease [22]. The resultant increase in microvascular density (MVD) is associated with higher tumour grade and worse prognosis [22]. The increased perfusion in malignant tissue is targeted by Doppler ultrasound imaging. Colour Doppler ultrasound (CDU) depicts flow by exploiting the shift in frequency that occurs when the signal is reflected by blood cells that are moving away or towards the transducer [22]. Power Doppler ultrasound (PDU) is more sensitive but does not depict the direction of flow. PDU can detect flow in vessels as small as 1 mm and therefore allows visualization of a tumour’s feeding vessels. However, the true angiogenic microvessels are in the 10–50 μM range [22].

Various authors reported additional value of the Doppler techniques over GSU [8, 10, 13]. In particular, two studies that reconstructed the vascularization in 3D with PDU performed well [8, 12]. The largest study to date, by Eisenberg et al. [9], compared GSU and PDU with 620 RP specimens and reported that adding PDU to GSU improved specificity from 47 to 74 %, although the sensitivity decreased from 58 to 47 %. Results vary greatly between studies, reflecting differences in study design, imaging protocols and population. A meaningful additional benefit of these techniques compared to GSU within each study could not be universally demonstrated [23, 24]. As hypervascularity detected by Doppler ultrasound is not based on true angiogenic microvessels but on the larger feeding vessels, Doppler US is more sensitive to larger, higher Gleason grade lesions [10, 13].

### Contrast-enhanced ultrasound

In contrast-enhanced ultrasound, an ultrasound contrast agent (UCA) consisting of gas-filled microbubbles is administered intravenously just prior to or during ultrasound imaging [25]. The microbubbles have diameters comparable to that of erythrocytes, enabling them to pass the microvasculature [22]. Contrast ultrasound has been used to evaluate perfusion of the heart and abdominal organs [26, 27].

The microbubbles were first used as additional reflectors in combination with the Doppler techniques, supposedly increasing sensitivity. Sedelaar et al. [28] first demonstrated that MVD, associated with PCa, was almost double in areas that presented contrast-enhanced power Doppler ultrasound (CE-PDU) enhancement compared to unenhanced prostate areas. They were able to find an average of 86 % of PCa’s in 70 patients scheduled for RP using 3D CE-PDU [29]. A recent, large trial by Mitterberger et al. [30] comparing the detection rates of five contrast-enhanced colour Doppler ultrasound (CE-CDU)-targeted biopsies and ten systematic biopsies in 1776 men found a significantly higher positive core rate for the targeted biopsies compared to the systematic biopsies (11 vs 5 %). A smaller biopsy-based study by Taverna et al. [24] was not able to demonstrate a significantly higher positive biopsy rate of CE-PDU compared to PDU or GSU.

**Fig. 2:**
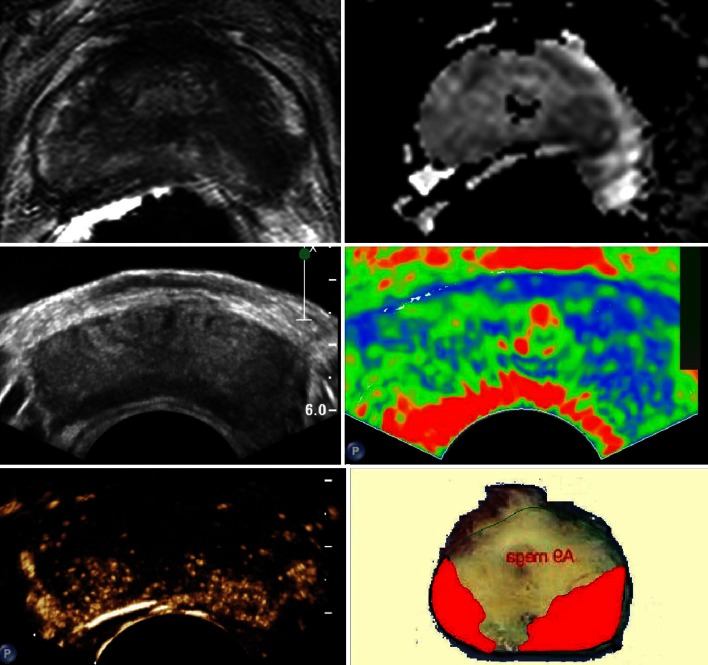
Multiparametric ultrasound and MRI modalities. *Top left *and *right* T2-MRI and diffusion-weighted MRI indicating tumour presence on the *left* side. *Middle left *and *right* GSU and elastography indicating tumour presence on the *left* side. *Bottom left *and *right* DCE-US and pathology indicating tumour presence on both sides

In the past years, dynamic contrast-enhanced ultrasound (DCE-US) has emerged, using low-energy US pulses preventing the premature bursting of the microbubbles [31]. This technique exploits the microbubbles’ nonlinear oscillations in the ultrasound field, causing nonlinear reflections which can be discriminated from the linear tissue reflections [31]. This allows contrast-specific imaging, sensitive enough to detect a single microbubble and therefore visualization of blood flow through the true microvasculature [32]. Several features are associated with malignancy: asymmetrical rapid inflow (enhancement), increased focal enhancement and asymmetry of intraprostatic vessels (Fig. 2) [33]. A limited number of studies have compared DCE-US imaging with RP specimens. Halpern et al. [34] and Matsumoto et al. [35] achieved sensitivities of 42 and 41 % combining DCE-US and GSU in 12 and 50 patients, respectively. Unfortunately, their study design did not allow calculation of specificity. More recent studies are presented in Table 1. A yet unpublished study from our own institution compared the diagnostic performance parameters of DCE-US and mpMRI in 36 patients using RP specimens as reference standard. For lesions 0.5 mL or larger, the two observers for DCE-US achieved a sensitivity of 58–69 % and a specificity of 93–95 % which was comparable to the performance of mpMRI under the same conditions. Qi et al. [36] were able to detect the index tumour in 67 of the 83 patients they examined with DCE-US before RP.

**Table 1:** Overview of studies assessing diagnostic performance of transrectal ultrasound-based imaging modalities for the detection of prostate cancer

Modality	Author	Patients	Design	Sens.	Spec.	PPV	NPV
GSU	Sauvain [8]	282	GSU versus 6–8 biopsies	88	58	72	79
	Eisenberg et al. [9]	620	GSU versus RP	59	47	91	11
	Zalesky et al. [12]	146	GSU versus RP	8	99	83	59
	Kuligowska et al. [10]	544	GSU versus 12 biopsies	41	85	53	72
C-TRUS	Walz et al. [16]	28	C-TRUS versus RP	83	64	80	68
	Loch et al. [18]	164	C-TRUS versus RP	82	80		
Doppler	Sauvain [8]	282	3D-PDU versus 6–8 biopsies	92	72	80	88
	Kuligowska et al. [10]	544	CDU versus 12 biopsies	53	66	41	69
	Eisenberg et al. [9]	620	PDU versus RP	40	35	88	6
	Zalesky et al. [12]	146	3D-PDU versus RP	62	79	72	70
DCE-US	Seitz et al. [33]	35	DCE-US versus RP and RCP	69	33	84	18
	Unpublished data from AMC	36	DCE-US + GSU versus RP	58–69	93–95		
	Unpublished data from AMC	11	Semiquantative DCE-US + GSU versus RP	87	84		
	Jung et al. [41]	20	Semiquantitative DCE-US versus RP	88	100		
SE	Zhang et al. [44]	508	Meta-analysis of 7 studies: SE versus RP	72	76		
	Teng et al. [45]	527	Meta-analysis: SE-targeted biopsy versus systematic biopsy	62	79		
SWE	Ahmad et al. [49]	50	Per ROI SWE versus 12 biopsies	90–93	88–93	93–98	83–81
	Barr et al. [48]	53	Per ROI SWE versus 12 biopsies	96	96	69	100

Performance parameters in percentages rounded to integers

*GSU* greyscale ultrasound, *RP* radical prostatectomy, *C*-*TRUS* computer-assisted transrectal ultrasound, *DCE*-*US* dynamic contrast-enhanced ultrasound, *SE* strain elastography, *SWE* shear wave elastography, *3D*-*PDU* three-dimensional power Doppler ultrasound, *RCP* radical cystoprostatectomy, *ROI* region of interest, *Sens*. sensitivity, *Spec.* specificity, *PPV* positive predictive value, *NPV* negative predictive value, *AMC* Academic Medical Center, Amsterdam, The Netherlands

Recently, quantification techniques are being developed that extract blood flow parameters from DCE-US data that help predict whether tissue is malignant using different blood flow models. Quantitative maps of perfusion parameters can be generated by analysis of the time evolution of the UCA concentration [37]. The latest developments are, however, focussing on the assessment of the dispersion kinetics of the UCA passing through the microvasculature as a better indicator of microvascular architecture and highlighting those changes related to cancer neoangiogenesis [38–40]. Quantification is thought to be able to improve accuracy and decrease user dependency associated with DCE-US interpretation [32]. Still to be published data from our own institution showed that using dispersion-based quantification increased sensitivity from 72 to 87 %, while specificity decreased from 92 to 84 % using 11 RP specimens as reference standard. Jung et al. [41] achieved a 88 % sensitivity and a 100 % specificity using DCE-US quantification in 20 patients scheduled for RP.

A major limitation in current DCE-US imaging is that recording the inflow and outflow takes about 2 min and only one plane can be recorded at a time. An interval of 3–5 min between boluses is required to allow sufficient UCA breakdown to be able to evaluate contrast inflow. A major step will be the use of 3D endorectal probes suitable for DCE-US. This will allow repetitive 3D (or 4D) scanning of UCA flow through the entire prostate after a single bolus injection. Availability of ultrasound systems with DCE-US compatible endorectal probes is limited, and no data on 4D DCE-US are published yet.

### Elastography

Most PCas are harder than normal prostatic tissue, which is the basis for PCa detection by DRE. This stiffening is caused by increased cellularity and microvascularity and a stromal response causing increased collagen deposition around the tumour [42]. Two variants of ultrasound elastography exploit this difference in stiffness: the more extensively evaluated quasi-static or strain elastography (SE) and the novel shear wave elastography (SWE).

In SE, cyclic compression is applied to the prostate using the endorectal probe, resulting in a changed morphology of the prostate tissues. Harder tissues are less affected than softer tissues, and this variation in the amount of deformation (strain) is displayed in the form of a colour overlay (Fig. 2) [43]. A recently published meta-analysis of seven studies comparing SE with RP specimens found a pooled sensitivity and specificity of 72 and 76 %, respectively [44]. A 2012 meta-analysis by Teng et al. [45] evaluating the accuracy of SE-targeted biopsies pooled the data of six studies to find a per-patient sensitivity of 62 %, specificity of 79 % and a per-core sensitivity and specificity of 34 and 93 %, respectively. Both meta-analyses confirm that heterogeneity in study designs exists, making direct comparison of results difficult. Nevertheless, SE has shown consistently good results with a clear additional value to GSU. Several authors found that higher Gleason grade lesions were more easily detected by SE [13, 46]. The subjective interpretation of the colour maps and the free-hand cyclic compression of the prostate add considerable user dependency to SE imaging. Tsutsumi et al. [47] improved their SE results by using balloon inflation to exert force on the prostate rather than manual compression. A major drawback of SE is that the colour map is automatically scaled between the softest and the hardest tissue in the imaged field. It contains no absolute measure of elasticity, disabling quantification techniques and comparison of stiffness between patients [43].

SWE is a novel technique that assesses stiffness by measuring the velocity at which a shear wave travels through the tissues. The shear wave, induced within the tissue by using the acoustic radiation force produced by a focused ultrasound beam, propagates faster through stiffer tissue [48]. The speed of shear wave propagation is related to the Young’s modulus (the ratio of stress put on the tissue to the resulting deformation) and is displayed as a colour map [43]. Two clear advantages are that SWE does not require manual cyclic compression of the prostate and quantification is possible because shear wave velocity and Young’s modulus are absolute values. Only a limited amount data are available for SWE. Correlating SWE imaging and sextant biopsy results in 53 men, Barr et al. [48] achieved a sensitivity of 96 %, a specificity of 96 %, a PPV of 69 % and a NPV of almost 100 %. Young’s modulus was significantly higher in areas with malignant tissue compared to areas with atypia or inflammation. In a study with a similar design among 50 patients by Ahmad et al. [49], SWE reached sensitivities and specificities of 90 and 88 % in patients with a PSA value below 20 ng/mL and 93 and 93 % in patients with a PSA value above 20 ng/mL. Their data also suggest a relationship between Young’s modulus and Gleason grade.

### mpUS

The ultrasound modalities discussed here exploit different physical characteristics of malignant tissue: GSU and C-TRUS visualize anatomical structures; the Doppler techniques depict increased macrovascularity, DCE-US targets microvascularity, and elastography the increased stiffness. Therefore, combining the modalities has the potential to detect more tumours while being more specific because more characteristics of suspicious lesions are evaluated (Fig. 2). Unfortunately, there are limited data on the performance of combinations of ultrasound modalities. A study in 133 patients by Aigner et al. [50] showed that a median of five biopsies targeted at SE and DCE-US lesions resulted in a higher per-patient detection rate (59.4 %) than what can be expected of systematic biopsies. Unfortunately, their study design does not allow calculation of false negatives. The systematic literature search identified two additional studies using systematic biopsy as a reference standard and only one study correlating mpUS with RP specimens (Fig. 1). Nelson et al. [13] compared GSU, PDU and SE in 137 patients using targeted biopsies with sextant systematic biopsies as reference standard. Of the 106 positive biopsy sites, GSU was positive in 16 %, CDU in 29 %, SE in 25 % and the combination in 46 %, showing that the three modalities detect different tumours. Xie et al. [51] compared the results of a 10 core biopsy scheme with GSU, PDU and DCE-US imaging in 150 patients. GSU was positive in 51 % of cancer sites, PDU in 48 % and DCE-US in 73 %, while the combination was positive in 82 % of cancer sites. Both studies combine the ultrasound modalities by adding up all lesions seen by any of the modalities. This by default increases sensitivity and NPV at the cost of specificity and PPV. Unfortunately, insufficient data were supplied in the articles to calculate specificity for the combinations. Conversely, other authors combine ultrasound modalities by using the second modality to further characterize lesions highlighted by the first. This will produce a higher specificity and PPV at the cost of sensitivity and NPV. Brock et al. [52] compared SE and DCE-US with 100 RP specimens. Prostates were scanned with SE, resulting in a sensitivity of 49 % and a specificity of 74 %. Eighty-six target lesions were defined by SE and further characterized by DCE-US. This way the PPV of the target lesions increased from 65 % for SE alone to 90 % for the combination of SE and DCE-US.

A clinically more valuable balance between sensitivity and specificity or NPV and PPV can be obtained by the methods described above. When cancer detection is considered paramount, additional ultrasound modalities pinpointing more suspicious areas for targeted biopsy might prove usable if the combined NPV is high and the number of targeted biopsies workable. The studies by Nelson et al. and Xie et al. show that a 13–59 % increase in sensitivity can be achieved by combining the better performing modality with a second lower performing modality in a crude fashion. A potentially better system for combining imaging modalities would be a scoring system analogous to the mpMRI’s PI-RADS. This system uses Likert-type scales for each modality and a total score representing the chance of cancer presence for that region [53]. However, a more sophisticated classifier algorithm, fully exploiting and integrating the complementary qualities of the different imaging modalities, could be constructed based on, for example, a support vector machine or an artificial neural network [54]. This would allow incorporation of human interpreted imaging data on Likert-type scales, unstratified quantification data and even patient characteristics. Ideally, the output of such an algorithm should differentiate between high-risk and low-risk disease. Prerequisites for the development of such an advanced mpUS system are the acquisition of data in large patient cohorts using standardized imaging protocols.

## Discussion

This review shows that promising results can be achieved by ultrasound-based imaging (Table 1). Sensitivities approach many of those reported for mpMRI and surpass those of the individual modalities that constitute mpMRI [55–58]. Our literature search shows that small steps have been undertaken in combining ultrasound techniques and that these have led to improved results. Larger (multicentre) trials with standardized imaging protocols and reporting are necessary to reduce user dependency and get more accurate estimates of the sensitivity and specificity of the individual ultrasound modalities. A standardized reporting system for ultrasound modalities is therefore urgently needed. Subsequently, refined methods of combining the modalities need to be developed and validated. In order to validate the mpUS as a whole or any of the modalities involved, a reliable method of correlating histopathology to imaging is needed. Methodological challenges including mismatch between imaging and pathology have complicated research and contributed greatly to the varying results on both the MRI and ultrasound platforms. Authors have tried to compensate for mismatch between histopathology and imaging, but one can never be certain whether over- or under-compensation arises [56, 57]. Turkbey et al. [58] devised a method using prostate-specific 3D-printed molds to eliminate plane angulation problems. 3D registration of histopathology and imaging as proposed by Mischi et al. [59] also corrects for plane mismatch and could accommodate multiple imaging modalities to be mapped with histopathology.

Although mpMRI results from expert centres are encouraging, these results vary widely and could not be confirmed in clinical practice yet. Further research is needed to finally establish consistency [56, 58]. In addition, the mpMRI’s PI-RADS system that is used widely has not been validated in multicentre studies as suggested in the original article by the authors. Because of the influence of methodology on results, the only reliable way to compare the performance of ultrasound techniques with each other or with mpMRI is to perform both in the same study on the same patient cohort. Yet unpublished results from our own institution showed that DCE-US performed equal to mpMRI in such a direct comparison. Several other factors are important in the debate around the positioning of MRI and ultrasound in clinical practice. The widespread usage of mpMRI is prevented by issues of availability, costs and technical challenges associated with taking biopsies in high magnetic fields [60]. For now, in most centres, mpMRI is reserved for selected patients with repeated prior negative biopsies and persistent clinical suspicion for malignancy [1]. Ultrasound-based imaging shares with MRI the advantage of not using ionizing radiation; therefore, repeated imaging can be performed without harming the patient. Major advantages of ultrasound over MRI are a wider availability, reduced cost and time consumption, and the fact that it can be performed by the urologist, in the office setting or at the bedside.

## Conclusion

Important advances have been made in the development of ultrasound-based imaging modalities. The latest clinical results achieved using these techniques achieve excellent results that need to be matched by other modalities. Data from recent advances in the form of contrast-compatible 4D transrectal probes and quantification techniques for GSU, C-TRUS, DCE-US and SWE are expected to show improved results in the near future. By effectively combining these ultrasound techniques, all targeting different properties of malignant tissue, a valuable clinical tool with all the advantages of ultrasound could be constructed. The literature shows that combining ultrasound modalities in a crude fashion can already improve sensitivity by 13–59 %. To unlock the full potential of mpUS, standardized imaging protocols and an optimal algorithm for combining the imaging results should be developed and validated. No research to date integrates the ultrasound modalities in such a way.
